# Established risk prediction models for the incidence of a low lean tissue index in patients with peritoneal dialysis

**DOI:** 10.1080/0886022X.2022.2113794

**Published:** 2022-08-29

**Authors:** Feng Li, Lei Wang, Yanling Mao, Changqing Mao, Jie Yu, Dan Zhao, Yingying Zhang, Ying Li

**Affiliations:** a Department of Nephrology, Jiading District Central Hospital Affiliated Shanghai University of Medicine & Health Sciences, Shanghai, China; b Department of Nephrology, Tongji Hospital, School of Medicine, Tongji University, Shanghai, China

**Keywords:** Peritoneal dialysis, lean tissue index, malnutrition, bioimpedance spectroscopy, subjective global assessment

## Abstract

**Objective:**

The objective of this study is to investigate the incidence of low lean tissue index (LTI) and the risk factors for low LTI in peritoneal dialysis (PD) patients, including to establish risk prediction models.

**Methods:**

A total of 104 PD patients were enrolled from October 2019 to 2021. LTI was measured by bioimpedance spectroscopy. Multivariate logistic regression and machine learning were used to analyze the risk factors for low LTI in PD patients. Kaplan–Meier analysis was used to analyze the survival rate of patients with low LTI.

**Results:**

The interleukin-6 (IL-6) level, red cell distribution width (RDW), overhydration, body mass index (BMI), and the subjective global assessment (SGA) rating significantly differed between the low LTI and normal LTI groups (all *p* < 0.05). Multivariate logistic regression showed that IL-6 (1.10 [95% CI: 1.02–1.18]), RDW (1.87 [95% CI: 1.18–2.97]), BMI (0.97 [95% CI: 0.68–0.91]), and the SGA rating (6.33 [95% CI: 1.59–25.30]) were independent risk factors for LTI. Cox regression analysis showed that low LTI (HR 3.14, [95% CI: 1.12–8.80]) was the only significant risk factor for all-cause death in peritoneal dialysis patients. The decision process to predict the incidence of low LTI in PD patients was established by machine learning, and the area under the curve of internal validation was 0.6349.

**Conclusions:**

Low LTI is closely related to mortality in PD patients. Microinflammatory status, high RDW, low BMI and low SGA rating are risk factors for low LTI in PD patients. The developed prediction model may serve as a useful tool for assessing low LTI in PD patients.

## Introduction

Peritoneal dialysis (PD) is an important alternative treatment for kidney failure [1]. Malnutrition is one of the most common complications for PD patients, and studies have shown that the incidence of malnutrition is between 18% and 75% [2]. Malnutrition is closely related to the quality of life, incidence of peritonitis and mortality of PD patients [3,4]. The Lean Tissue Index (LTI), based on bioelectrical impedance analysis, is a new technique for evaluating the nutritional status of patients [5–7]. Recent studies have found that low LTI is an independent risk factor for cardiovascular death and all causes of death in kidney failure patients [8]. Early diagnosis and intervention using low LTI are important approaches for improving the quality of life and survival rate of kidney failure patients. Rymarz et al. [5] found that a low LTI in hemodialysis patients was closely related to age and the concentrations of interleukin-6 (IL-6) and insulin-like growth factor-1. However, the incidence of low LTIs in PD patients and its influencing factors have rarely been reported. In this study, multifrequency bioelectrical impedance was used to measure the LTI in PD patients. Traditional statistics and machine learning were used to analyze the incidence of low LTI and related influencing factors. The establishment of an LTI prediction model provided a theoretical basis for the further early identification of low LTI in PD patients. Overall, this study aimed to explore the independent risk factors for low LTI in PD patients and develop predictive models using traditional statistical methods and machine learning techniques.

## Methods and materials

### Patients

Patients who were receiving regular PD between October 2019 and 2021 at Shanghai Jiading District Central Hospital, who were >18 years old and had received PD for ≥3 months were enrolled in the study. The exclusion criteria were age <18 years; confirmed diagnosis of hematological diseases, such as multiple myeloma, acute infectious disease, malignant tumors, cirrhosis of the liver, amputation, the presence of metal stents or pacemakers (which could interfere with bioelectrical impedance measurements), and incomplete data (Supplementary Figure S1). The study was approved by the Ethics Committee of Shanghai Jiading District Central Hospital, and all patients signed informed consent (Ethics No. 2019K08). All patients in the current study were prescribed continuous ambulatory peritoneal dialysis (CAPD). The dialysis regimen was as follows: dialysate glucose concentration of 1.5% or 2.5% (Dianeal^®^, Baxter), abdominal retention duration of 4–6 h, and dialysate volume of 6000–10,000 mL/day.

### General data collection

The demographic data of enrolled patients collected included sex, age, dialysis history, height, weight, primary disease, complications and other factors. The medical history of cardiovascular disease included previous angina pectoris, myocardial infarction, congestive heart failure, coronary artery bypass grafting or stenting, old cerebral infarction, and peripheral vascular disease [9]. The brachial artery blood pressure of the right arm was measured 3 consecutive times to obtain the average pressure after 15 min of rest for all patients. The nutritional status of patients was evaluated using the 7-point subjective global assessment (SGA) scale, which contained medical history and physical examination [10]. The medical history consisted of four categories: weight loss, gastrointestinal symptoms, functional capacity, and comorbidities. The physical examination included a loss of subcutaneous fat, muscle wasting, and edema. Each component was rated from 1 to 7, and the overall SGA score was determined. Based on the overall SGA score, patients were classified into three groups: A = SGA score 6–7 (well nourished), B = SGA score 3–5 (mildly to moderately malnourished), or C = SGA score 1–2 (severely malnourished). None of the patients fit the criteria for Group C (severely malnourished) in our study. Body mass index (BMI) was calculated as follows: BMI = body mass (kg)/height (m^2^), and the Mosteller Formula [11] was used to calculate body surface area. Moreover, previous studies reported that the Charlson score was related to LTI. Thus, we calculated this score using the medical history reported by the patient, cited in the medical record, or detected during the medical examination [12].

### Biochemical index detection

Fasting venous blood samples were collected from all patients. Hemoglobin and the red cell distribution width (RDW) were measured by a Japanese SYSMEX XN-1000 automatic blood routine detector. An ABBOTT Architect C16000 Automatic dry chemical analyzer was used to detect the serum albumin (sAlb), prealbumin (pre-Alb), glucose, serum creatinine (sCr), triglyceride (TG), total cholesterol (TC), high-density lipoprotein cholesterol (HDL-C), and low-density lipoprotein cholesterol (LDL-C) concentrations. The adjusted calcium (Ca) levels were calculated with the following formula. Adjusted Ca = serum Ca + 0.02×(40-sAlb). An ABBOTT Architect I2000SR automatic chemiluminescence immunoassay was used to measure intact parathyroid hormone. The high-sensitivity C-reactive protein level was determined with a Beckman Array 360 System using scattering rate turbidimetry. The serum IL-6 level was measured by ELISA.

### PD-related indicators

Dialysis prescriptions, 24-h urine, and ultrafiltration volumes were recorded. A standard peritoneal balance test was performed to determine the dialysate creatinine/serum creatinine ratio at 4 h (4 hD/Pcr), as previously reported [13]. The weekly total urea clearance index (Kt/V), total creatinine clearance rate (CCr), and normalized protein catabolic rate were calculated. The arithmetic mean value of creatinine and urea clearance was used to calculate the residual renal function (RRF) and daily glucose exposure in the dialysate using the following equation:

Glucose exposure (g/day)=1.5% PD solution (mL)×1.5%+2.5% PD solution (mL)×2.5%+4.25% PD solution (mL)×4.25%

### LTI determination and grouping

The patients’ LTI and overhydration (OH) were measured using the Body Composition Monitor (BCM) based on multifrequency bioelectrical impedance technology (Fresenius Medical Care, German). The datasheet of the bioimpedance meter is available at https://www.freseniusmedicalcare.com/en/body-composition-monitor. Bioelectrical impedance spectroscopy (BIS) in the whole body (BISWB) was measured in this study [14]. All measurements were performed 2 h after the PD solution was retained in the abdomen, strictly following the operation manual. Data were exported using the Fluid Management Tool (ver. 3.3 English) provided by Fresenius Medical Care. The enrolled patients were divided into a low LTI group and a normal LTI group according to whether their LTI was below the 10th percentile of the reference range for healthy people of the same sex and age [6,15]. The LTI was measured by the same operator at least three times, and the two closest values were selected as the inclusion criteria.

### Statistical analysis

The SPSS (ver. 22.0) software was used for all statistical analyses. Quantitative data with a normal distribution are presented as $\bar{X}$ ± *s,* and quantitative data with a non-normal distribution are presented as Md (P_25_, P_75_). An independent sample *t* test and a Mann–Whitney *U* test were used to compare differences between groups. Qualitative data are given as cases (%) and compared using a χ^2^ test. Univariate logistic regression was used to analyze factors that influence a low LTI, and independent variables with *p* < 0.1 and clinically close relationships with dependent variables were screened. Multivariate stepwise logistic regression was used to analyze the independent risk factors for a low LTI. The test level was two-sided, and *p* < 0.05 was considered to be statistically significant. The Kaplan–Meier method was used to evaluate the survival curve, and Cox proportional hazard regression models were used to assess the risks of mortality for PD patients. Potential risk factors were evaluated using univariate logistic analysis, and the predictors (*p* < 0.15) were included in Cox regression analysis. Other inclusion factors were based on clinical experience, such as IL-6 levels, RDW, serum albumin levels, and OH.

### Machine learning

All included data were randomly sampled; according to conventional methods, 70% of the data were used as the training set for the machine learning model, and the remaining 30% were used as the set for testing the final performance of the model. This process was repeated five times, and the mean value was taken as the final result to weaken the influence of random partitioning and increase the stability of the result. Moreover, cross-validation was used to improve the accuracy of the models. Five classifiers, random forest (RF), gradient boosted decision tree (GBDT), decision tree (DT), gradient lifting (GBM), and support vector machine (SVM), were used to construct prediction models based on training data. Based on different machine algorithms, the area under the curve (AUC) was used to screen for the optimal model, determine important features and further build a visual DT and naive Bayes Model [16,17].

Python (ver. 3.6.5) was used for model building and performance evaluation. Data at baseline are expressed as $\bar{X}$ ± s. Other statistical methods were used to analyze comparisons between groups and whether the results fit a normal distribution. All data were directly incorporated into Python to evaluate logical relationships using various algorithms.

## Results

### Demographic characteristics of the enrolled patients

A total of 104 PD patients, including 61 males (58.7%), with an age range of 64.93 ± 10.78 years who had a dialysis history of 31.12 months (17.19, 53.8%) were included in the study. Among these 104 patients, 18 patients passed away, and no patients were transferred to HD or kidney transplantation during follow-up. The mean follow-up time was 21.00 (19.00–23.00) months, the minimum follow-up time was 3 months, and the maximum follow-up time was 24 months. The primary disease was chronic glomerulonephritis in 35 cases (33.65%), diabetic nephropathy in 39 cases (37.50%), polycystic kidney in 6 cases (5.77%), and hypertensive nephrosclerosis in 6 cases (5.80%); the cause was unknown in 18 cases (17.31%). Among the included cases, 51 cases (49.04%) were complicated by diabetes, 28 (26.92%) by cardiovascular disease, and 81 (77.88%) by hypertension. SGA rating was grade A in 85 cases (81.73%) and grade B in 19 cases (18.27%). The demographic and clinical data of the patients are shown in Table 1.

**Table 1.** Comparison of general clinical data between the two groups.

Characteristics	Total (*n* = 104)	Low LTI (*n* = 49)	Normal LTI (*n* = 55)	*p* Value
Age, years ($\bar{x}$±*s*)	64.93 ± 10.78	63.16 ± 9.13	66.50 ± 11.93	0.12
Male, *n* (%)	61 (58.65)	27 (55.10)	34 (61.82)	0.49
PD length (month)	31.12 (17.19–53.83)	39.53 (23.15–57.97)	29.37 (13.27–52.07)	0.15
BMI (kg/m^2^)	24.70 (21.10–26.75)	22.80 (20.90–25.50)	25.60 (22.90–27.80)	**0.01**
BSA (m^2^, $\bar{x}$±*s*)	1.71 ± 0.18	1.68 ± 0.18	1.73 ± 0.19	0.17
Complications, *n* (%)				
DM	51 (49.04)	25 (51.02)	26 (47.27)	0.70
CVD	28 (26.92)	17 (34.69)	11 (20.00)	0.09
HBP	81 (77.88)	37 (75.51)	44 (80.00)	0.58
Blood pressure (mmHg)				
SBP ($\bar{x}$±*s*)	148.15 ± 22.66	148.16 ± 24.83	148.15 ± 20.76	0.99
DBP	81.00 (76.00–85.75)	81.00 (78.50–89.00)	81.00 (75.00–84.00)	0.10
SGA rating				
Grade A	85 (81.73)	35 (71.43)	50 (90.91)	**0.01**
Grade B	19 (18.27)	14 (28.57)	5 (9.09)	
CCI	3.00 (2.00–4.00)	4.00 (2.00–4.00	3.00 (2.00–4.00)	0.21
Hb (g/L, $\bar{x}$±*s*)	104.37 ± 16.21	104.55 ± 18.15	104.20 ± 14.42	0.91
RDW (%)	13.60 (12.73–14.40)	13.90 (13.05–14.90)	13.00 (12.50–13.90)	**0.01**
sAlb (g/L)	33.00 (30.00–36.00)	33.00 (29.00–36.00)	33.50 (30.75–37.25)	0.61
Pre-alb (mg/L, $\bar{x}$±*s*)	307.83 ± 75.98	293.42 ± 66.61	320.66 ± 81.93	0.07
Glu (mmol/L)	6.80 (5.70–9.20)	7.20 (5.65–9.95)	6.50 (5.70–8.60)	0.55
sCr (μmol/L, $\bar{x}$±*s*)	879.25 ± 282.33	880.79 ± 281.23	877.88 ± 285.89	0.96
TG (mmol/L)	1.72 (1.08–2.10)	1.49 (1.05–2.09)	1.79 (1.15–2.12)	0.30
TC (mmol/L, $\bar{x}$±*s*)	4.54 ± 1.27	4.50 ± 1.33	4.59 ± 1.23	0.72
HDL-C (mmol/L)	0.94 (0.82–1.15)	0.93 (0.80–1.13)	0.97 (0.82–1.16)	0.55
LDL-C (mmol/L)	2.55 (1.79–3.40)	2.62 (1.70–3.28)	2.51 (1.96–3.44)	0.94
hs-CRP (mg/L)	1.17 (0.50–4.13)	1.03 (0.50–5.80)	1.19 (0.50–3.79)	0.96
IL-6 (ng/L, $\bar{x}$±*s*)	31.50 ± 7.71	33.93 ± 7.21	29.30 ± 7.55	**0.01**
Adjusted Ca (mmol/L)	2.45 (2.38–2.54)	2.48 (2.42–2.60)	2.44 (2.34–2.54)	0.07
P (mmol/L, $\bar{x}$±*s*)	1.44 ± 0.37	1.44 ± 0.39	1.44 ± 0.35	0.97
iPTH (pg/ml)	240.70 (128.38–396.00)	212.45 (111.60–452.25)	249.30 (141.28–378.53)	0.93

BMI: body mass index; BSA: body surface area; DM: diabetes mellitus; CVD: cardiovascular disease; HBP: high blood pressure; SGA: subject global assessment; CCI: Charlson comorbidity index; Hb: hemoglobin; RDW: red cell distribution width; sAlb: serum albumin; Glu: glucose; sCr: creatinine; TG: triglyceride; TC: total cholesterol; HDL-C: high density lipoprotein cholesterol; LDL-C: low density lipoprotein cholesterol; hs-CRP: high-sensitivity C-reactive protein; IL-6: interleukin 6; Ca: calcium; P: phosphor; iPTH: intact parathyroid.

Data are presented as the Md (P25, P75), unless indicated otherwise. The boldface fronts mean *p* vaule considered statistically significant.

### Incidence of low LTI and comparison of clinical and laboratory indicators in different LTI groups

The low LTI group included 49 patients, and the incidence of low LTI in the enrolled patients was 47.1%. Compared with the normal LTI group, the BMI in the low LTI group was significantly decreased, and the SGA rating, RDW and IL-6 level were significantly increased (all *p* < 0.05) (Table 1). The results showed that BMI, SGA rating, RDW and IL-6 were tightly related to low LTI in PD patients.

### Comparison of PD-related indices and body composition in patients with different LTI groups

As shown in Table 2, compared with the normal LTI group, OH in the low LTI group was significantly higher (*p* < 0.05), but the total KT/V, total CCr, RRF, 24-h urine volume, or peritoneal transport mode did not significantly differ between groups. In addition to inflammation (IL-6), obesity (BMI), and malnutrition (SGA), overhydration contributed to low LTI.

**Table 2.** Comparison of PD-related indicators in patients with different LTIs.

	Total (*n* = 104)	Low LTI (*n* = 49)	Normal LTI (*n* = 55)	*p* Value
Glucose exposure (g/day)	130.00 (110.00–140.00)	140.00 (110.00–155.00)	120.00 (90.00–140.00)	0.17
Total Kt/V	1.65 (1.38–1.97)	1.64 (1.35–2.00)	1.68 (1.47–1.97)	0.56
Total CCr (mL/min)	46.09 (38.39–65.96)	44.66 (36.92–56.91)	50.76 (38.49–75.09)	0.18
RRF	0.72 (0–3.45)	0 (0–2.32)	1.06 (0–4.02)	0.11
nPCR [g/ (kg·day)]	0.67 (0.60–0.78)	0.66 (0.61–0.75)	0.68 (0.57–0.80)	0.60
4 h D/Pcr	0.58 (0.51–0.67)	0.58 (0.49–0.67)	0.61 (0.53–0.68)	0.19
24 h urine(ml)	300.00 (0–675.00)	0 (0–500.00)	400.00 (0–1000.00)	0.08
Peritoneal transport function, *n* (%)				
H	5 (4.81)	3 (6.12)	2 (3.64)	0.28
HA	23 (22.12)	10 (20.41)	13 (23.64)	
LA	20 (19.23)	13 (26.53)	7 (12.73)	
L	56 (53.84)	23 (46.94)	33 (60.00)	
OH (L)	3.00 (1.93–4.68)	3.80 (2.10–4.80)	2.80 (1.80–3.70)	0.04

Kt/V: urea clearance index; CCR: creatinine clearance rate; RRF: residual renal function; PCR: protein catabolic rate; H: high transport; HA: high average transport; LA: low average transport; L: low transport; OH: overhydration.

Data are represented as Md (P25, P75), unless indicated otherwise.

### Independent risk factors for low LTI in PD patients

A univariate logistic regression analysis revealed that BMI, the SGA rating, RDW, IL-6 level, and OH were factors related to a low LTI. After adjusting for sex, age, pro-albumin, calcium, OH, Kt/V, and other factors, multivariate logistic regression analysis showed that IL-6 (OR: 1.10, 95% CI: 1.02–1.18, *p* = 0.01), RDW (OR: 1.87, 95% CI: 1.18–2.97, *p* = 0.01), BMI (OR: 0.97, 95% CI: 0.68–0.91, *p* = 0.01), and the SGA rating (OR: 6.33, 95% CI: 1.59–25.30, *p* = 0.01) were independent correlation factors for a low LTI (Table 3).

**Table 3.** Logistic regression model analysis results of risk factors associated with a low LTI in PD patients.

Variables	Univariate analysis			Multivariate analysis		
	OR	95% CI	*p* Value	OR	95% CI	*p* Value
Age (years)	0.97	0.94–1.01	0.12	–	–	–
Male	0.79	0.35–1.69	0.49	–	–	–
PD length	1.01	0.99–1.03	0.12	–	–	–
BMI (kg/m^2^)	0.83	0.73–0.94	0.01	0.79	0.68–0.91	0.01
DM	1.16	0.54–2.51	0.70	–	–	–
SGA rating	4.00	1.32–12.12	0.01	6.33	1.59–25.30	0.01
DBP	1.03	0.99–1.07	0.12	–	–	–
Hb (g/L)	1.00	0.98–1.03	0.91	–	–	–
sAlb (g/L)	0.98	0.90–1.06	0.59	–	–	–
Pre-alb (mg/L)	0.99	0.99–1.00	0.07	–	–	–
hs-CRP (mg/L)	1.01	0.99–1.04	0.32	–	–	–
IL-6 (ng/L)	1.09	1.03–1.16	0.01	1.10	1.02–1.18	0.01
RDW (%)	1.90	1.29–2.81	0.01	1.87	1.18–2.97	0.01
RRF	0.88	0.75–1.03	0.10	–	–	–
Adjusted Ca (mmol/L)	5.75	0.79–41.86	0.08			
OH(L)	1.24	1.01–1.53	0.04	–	–	–
Kt/V (L/wk/1.73 m^2^)	0.82	0.36–1.85	0.63			

BMI: body mass index; BSA: body surface area; DM: diabetes mellitus; SGA: subject global assessment; DBP: diastolic blood pressure; Hb: hemoglobin; sAlb: serum albumin; hs-CRP: high-sensitivity C-reactive protein; IL-6: interleukin 6; RDW: red cell distribution width; RRF: residual renal function; OH: overhydration; Kt/V: urea clearance index.

A multifactor logistic regression analysis model was adjusted for gender, age, pro-alb, calcium, and OH.

### Machine learning analysis of the risk factors for a low LTI

**Figure 1.**
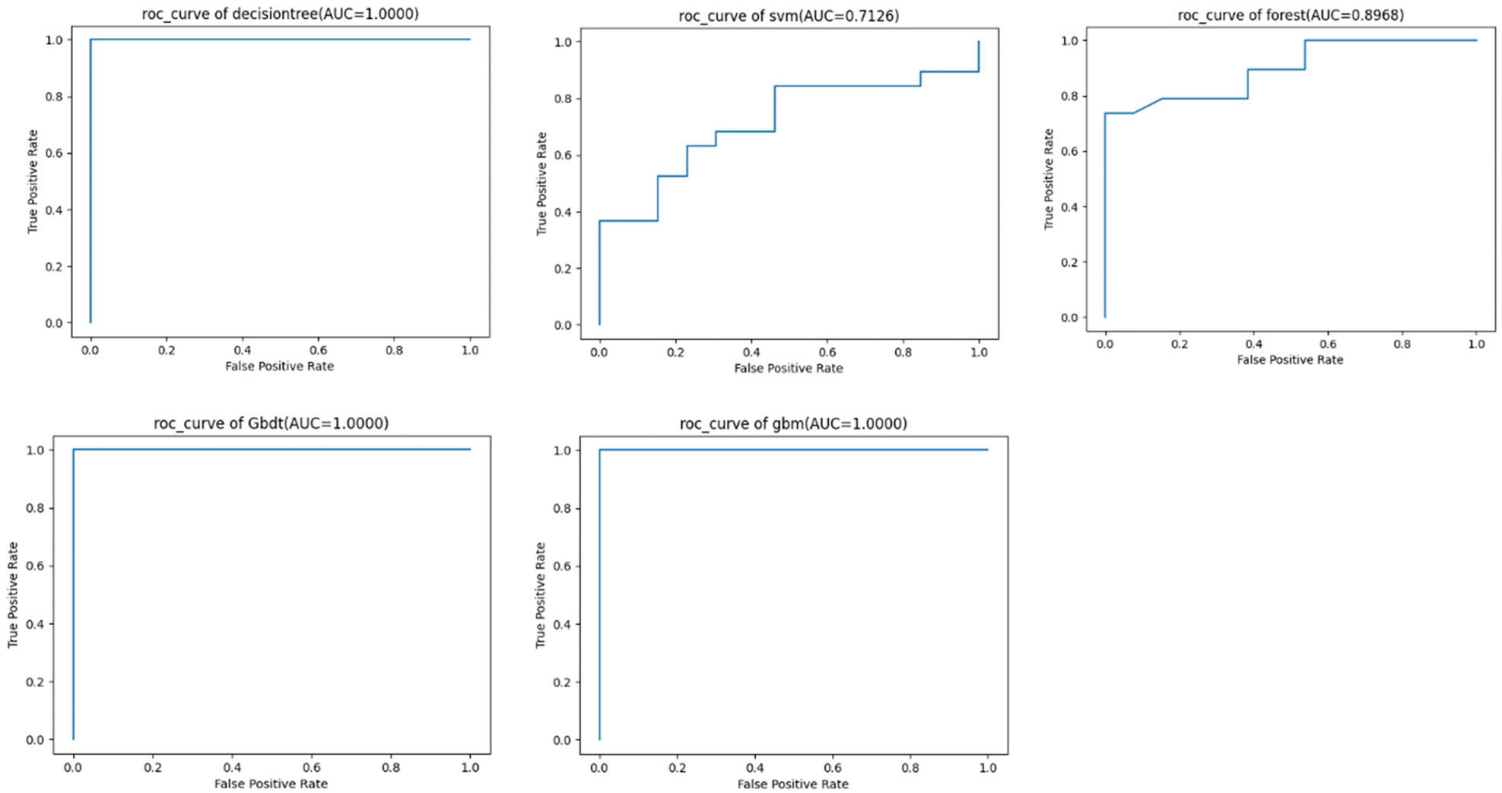
The ROC curves of each machine learning model.

RF, GBDT, DT, GBM, and SVM were used to train the data. Among these models, the performance of three models (DT, GBDT, and GBM) showed a better F1 score and AUC, suggesting good efficiency and good data quality (Table 4). Ten high-risk factors affecting the occurrence of low LTI were screened, including (1) nutritional status assessment indicators [adipose tissue mass (ATM), Charlson score, and BMI]; (2) PD-related indicators (24 h ultrafiltration, peritoneal Kt/V); (3) inflammation-related indicators (IL-6, neutrophil percentage); (4) cardiac function index [ejection fraction (EF)%, brain natriuretic peptide (BNP)]; and (5) others: age and RDW (Figure 1). The risk factors obtained by machine learning were consistent with those obtained by the SPSS software with regard to the inflammatory index, other indices, and the PD-related index. However, since machine learning analysis is black-box data analysis, missing data, and non-normally distributed data will not affect the analysis results. Therefore, according to the results obtained from SPSS, cardiac function and nutritional indicators are also high-risk factors affecting the occurrence of low LTI.

**Table 4.** The metrics of different machine learning models.

Model	Accuracy	Precision	Recall	F1 score	AUC
DT	1.000000	1.000000	1.000000	1.000000	**1.000000**
SVM	0.562500	0.777778	0.368421	0.500000	0.712551
RF	0.812500	0.882353	0.789474	0.833333	0.896761
GBDT	1.000000	1.000000	1.000000	1.000000	**1.000000**
GBM	0.968750	0.950000	1.000000	0.974359	**1.000000**

DT: decision tree; SVM: support vector machine; RF: random forest; GBDT: gradient boosted decision tree; GBM: gradient boosted machine; AUC: area under the curve. The boldface fonts mean that DT, GBDT and GBM show the best performance in terms of AUC values.

### Decision process analysis

**Figure 2.**
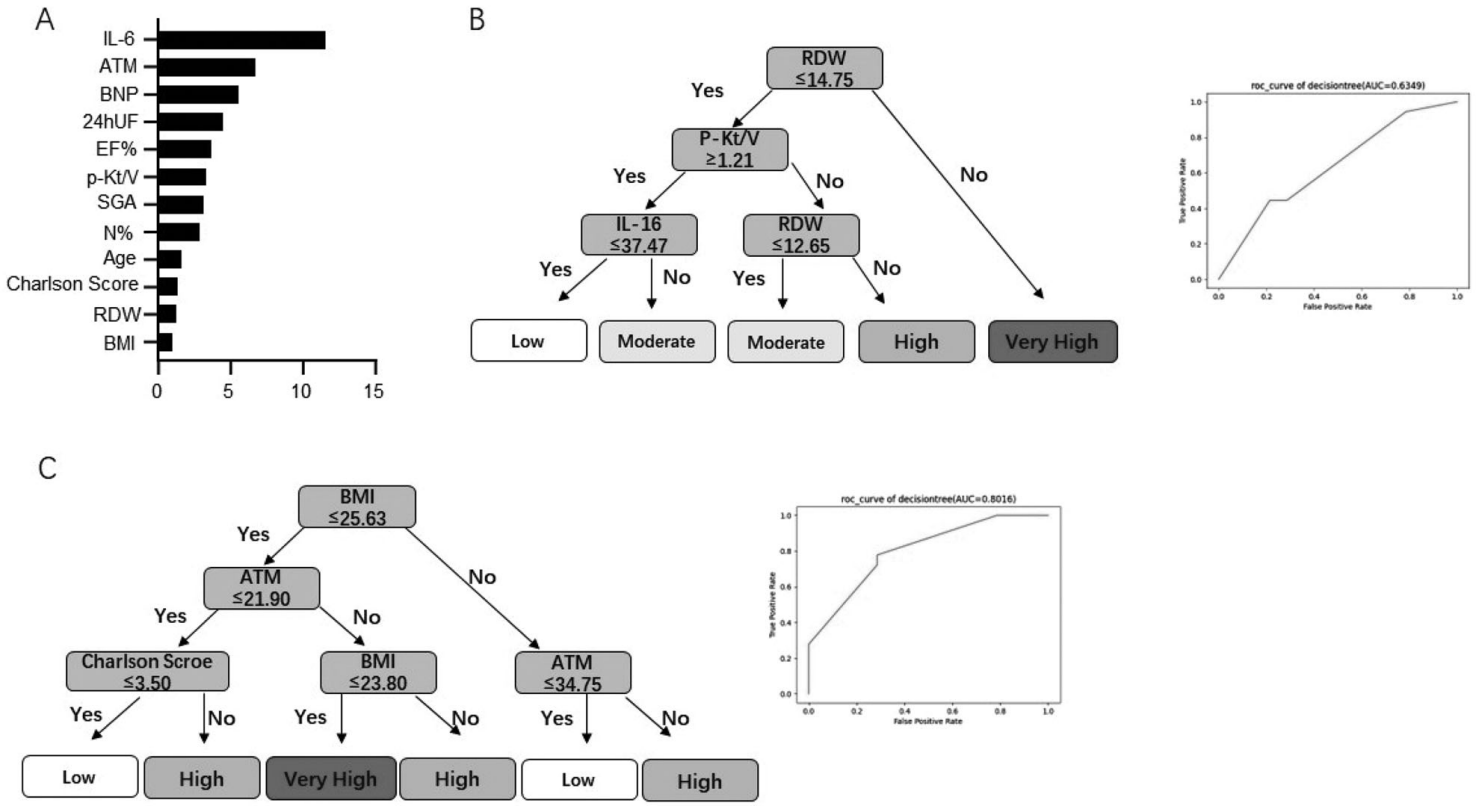
The feature importance of machine learning models and visual DT flow diagram. (A) The feature ranking analysis produced by the decision tree model. (B) A pruned decision tree flowchart for predicting low LTI incidence in PD patients. (C) A pruned decision tree flow chart based on nutrition-related indicators for predicting the risk stratification of low LTI incidence in PD patients. DT: decision tree; LTI: lean tissue index; PD: peritoneal dialysis.

Based on the univariate logistic regression analysis and machine learning screening, low LTI risk factors were divided into objective laboratory examination and subjective assessment factors, and LTI prediction models were constructed by the visual DT algorithm (Figure 2).Figure 2B shows the visual DT model based on objective indices, which can be divided into a four-layer model. After postpruning, RDW was found to be the primary factor affecting a low LTI in the important feature score of machine learning screening, while other indicators, including Kt/V, inflammation, and cardiac function-related indicators, were excluded due to the indirect influence. The AUC of the internal validation of the model was 0.6349. When nutrition-related indices were used to predict the incidence of low LTI (Figure 2C), the results suggested that the incidence was higher when the BMI and ATM indices were higher. However, LTI tended to be normal when the BMI and Charlson scores were low, which suggests that we can evaluate the risk of low LTI based on simple BMI, ATM, and Charlson scores in clinical practice. The AUC of internal validation of the model was 0.8016. The results showed that the two models listed above could predict the incidence of low LTI.

### Survival curve analysis

**Figure 3.**
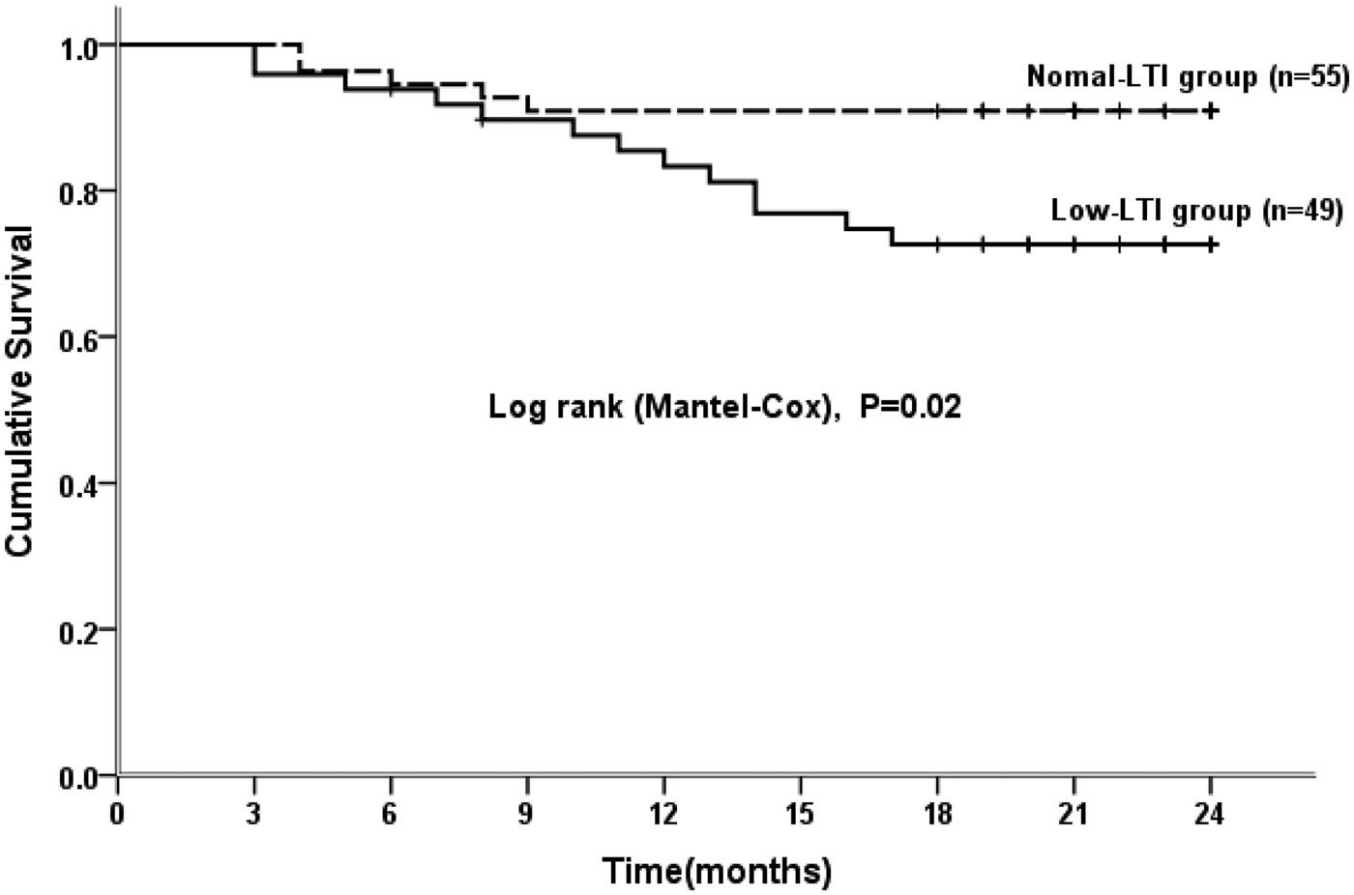
Kaplan–Meier survival curves of patients in the low LTI and normal LTI groups. LTI: lean tissue index.

Kaplan–Meier curve and Cox regression analyses were used to analyze the survival curve of patients with a low LTI, and the results indicated that for patients undergoing PD, the survival rate for those with a low LTI was markedly lower than that of patients with a high LTI (Figure 3). BMI, IL-6, RDW, low LTI, CVD history, serum albumin, OH, and Kt/V were included in the Cox analysis model. The Cox regression analysis showed that low LTI (HR 3.14, 95% CI: 1.12–8.80, *p* = 0.03) was the only significant risk factor for all-cause death in peritoneal dialysis patients after adjusting for other relevant factors (Table 5).

**Table 5.** Cox regression analysis of all-cause death in PD patients with low LTI.

Variables	Before correction			After correction		
	HR values	95% CI	*p* Values	HR values	95% CI	*p* Values
Age (years)	1.03	0.99–1.08	0.12			
Male	0.66	0.25–1.76	0.41	–	–	–
PD length (m)	1.00	0.99–1.02	0.59			
CVD history	2.14	0.83–5.52	0.12			
BMI (kg/m^2^)	0.90	0.79–1.03	0.12			
SGA rating	0.86	0.49–1.49	0.59			
IL-6 (ng/L)	1.01	0.95–1.07	0.84			
sAlb (g/L)	0.97	0.88–1.06	0.47			
RDW (%)	0.99	0.68–1.42	0.94			
Low LTI	3.17	1.13–8.90	0.03	3.14	1.12–8.80	0.03
OH (L)	1.10	0.90–1.34	0.36			
Kt/V(L/wk/1.73 m^2^)	0.75	0.27–2.11	0.59			

PD: peritoneal dialysis; CVD: cardiovascular disease; BMI: body mass index; SGA: subject global assessment; sAlb: serum albumin; IL-6: interleukin 6; RDW: red cell distribution width; LTI: lean tissue in; OH: overhydration; Kt/V: urea clearance index.

## Discussion

In this study, traditional statistical methods and machine learning were used to analyze the incidence of LTI in PD patients, the correlation between LTI and death, and the risk factors affecting low LTI. The results showed that PD patients with a low LTI generally had a low BMI, high microinflammatory state and a low peritoneal toxin clearance rate. Poor cardiac function, high RDW, and advanced age were factors affecting a low LTI, while a higher IL-6 level, RDW, and SGA rating and a low BMI were independent risk factors for a low LTI.

A significant finding of this study was that a low LTI was an important factor affecting the prognosis of PD patients. LTI assessed by multifrequency bioelectrical impedance technology is a new indicator for evaluating nutritional status [6] and has been confirmed by experts in China and abroad [6,8,15]. Although the European Working Group on Sarcopenia in Older People has proposed that a low LIT is one of the main diagnostic criteria for sarcopenia [18], Giglio et al. [19] showed that sarcopenia was closely related to the quality of life and hospitalization rate of elderly hemodialysis patients. However, the incidence of LTI in PD patients has not yet been reported to date. More importantly, our study showed that early diagnosis and intervention using low LTI were of great significance to improve the quality of life and survival rates of kidney failure patients.

The second major finding was the high-risk factors for low LTI patients identified using a traditional statistical method. The results of the traditional logistic regression analysis suggested that IL-6 level, SGA, BMI, and RDW were independent risk factors for a low LTI. The increase in IL-6 is a manifestation of microinflammatory activity. Serum proinflammatory cytokines, such as IL-6 and tumor necrosis factor α (TNF-α), can inhibit skeletal muscle differentiation and promote muscle decomposition by activating the adenosine triphosphate-ubiquitin–proteasome hydrolysis complex pathway or the nuclear factor-kappa B (NFκB) pathway [20]. RDW a routine test used to examine peripheral blood cells, and an increased RDW reflects the increased heterogeneity of RBC volume. In recent years, a high RDW level has been found to be common in dialysis patients. RDW was strongly associated with hospitalization rates and all-cause mortality in PD patients [21,22]. In addition, BMI and SGA rating are traditional indicators for evaluating the nutritional status of patients, which indirectly indicates that LTI is highly consistent with traditional nutritional assessment methods. As a simple and reliable method for evaluating malnutrition in PD patients, bioelectrical impedance technology warrants further promotion.

However, some data will be lost in a logistic regression analysis, e.g., non-normally distributed data. Incomplete clinical indicators with lost data could not be included in the study, and the internal relationship of data could not be evaluated. To compensate for the deficiencies highlighted above, the third important finding of our study was the utility of machine learning to supplement data analysis. The significant features were analyzed by machine learning based on blinding methods. To avoid insufficient sample-induced overfitting, cross validation and tree pruning methods were accessed in the study, which could improve the generalization and accuracy of the machine learning model. According to the machine learning results, the following risk factors affect the occurrence of a low LTI in PD patients: (1) nutritional status assessment indicators (ATM and BMI); (2) PD-related indicators (24 h ultrafiltration, peritoneal Kt/V); (3) inflammation-related indicators (IL-6 and neutrophil percentage); (4) cardiac function index (EF%, BNP); (5) and others: age and RDW. The above results suggested that the LTI ratio decreased in patients with a high ATM, and poor ultrafiltration on PD, microinflammation, poor cardiac function, and advanced age were risk factors affecting the occurrence of a low LTI in patients undergoing PD. The above results were also consistent with clinical practice findings. Nevertheless, we observed a few differences between the results of the logistic regression and those of the machine learning algorithm, such as overhydration and the Charlson score. In the machine learning algorithms, the results showed that overhydration could not predict low LTI incidence. The possible reason for this difference might be the limited sample size and one-to-one correspondence between numeric variables. Another disadvantage of machine learning is the repeatability of some parameters based on the blinding analysis.

Based on different statistical methods (logistics regression analysis and machine learning), we constructed a visual DT process to help clinicians predict the occurrence of LTI in patients receiving PD at an early stage through auxiliary examination and nutritional status, providing a theoretical basis for further early intervention.

The present study was subject to limitations. First, this study was cross-sectional in nature, and establishing a causal relationship between RDW, IL-6, SGA rating, and BMI in PD patients with low LT was consequently not possible. Second, the reference population of the low LTI group in our study was the healthy population in Europe and America, which may be affected by ethnic differences, diet structure and exercise levels. Third, most patients did not achieve the goal of sufficient dialysis (Kt/*V* > 1.7 L/wk/1.73 m^2^) in our PD center. According to previous studies, inadequate dialysis is a risk factor for malnutrition and might induce bias in the results [23]. Fourth, the sample size was insufficient, and external validation results were lacking. Finally, one disadvantage of multifrequency bioelectrical impedance analysis (BIA) is time consumption. Therefore, our potential future studies in collaboration with other centers, larger sample sizes and longer follow-up periods will hopefully address these limitations.

## Conclusions

Overall, PD patients have a high incidence of low LTI, and a low LTI is closely related to mortality after correcting for hyperhydration. Measuring LTI by bioimpedance meters seems to be noninvasive, simple and fast, which contributes to clinical work irrespective of overhydration status. Microinflammatory status, high RDW, peritoneal ultrafiltration toxin function and a low BMI are risk factors for a low LTI in PD patients. The development of the decision-making process will be a powerful tool for the early diagnosis and effective intervention of these patients in the clinic.

## Related research data

Source: Figshare, https://doi.org/10.6084/m9.figshare.19043786.

## Supplementary Material

Supplemental Material
