# Supplementary figures and images for: Established risk prediction models for the incidence of a low lean tissue index in patients with peritoneal dialysis
Source: Ren Fail. 2022 Aug 29;44(1):1417–25. doi: 10.1080/0886022X.2022.2113794 (PMC9448374; doi:10.1080/0886022X.2022.2113794)

**Supplementary Figure 1. Flow chart illustrating patient enrolment in the discovery cohort.**

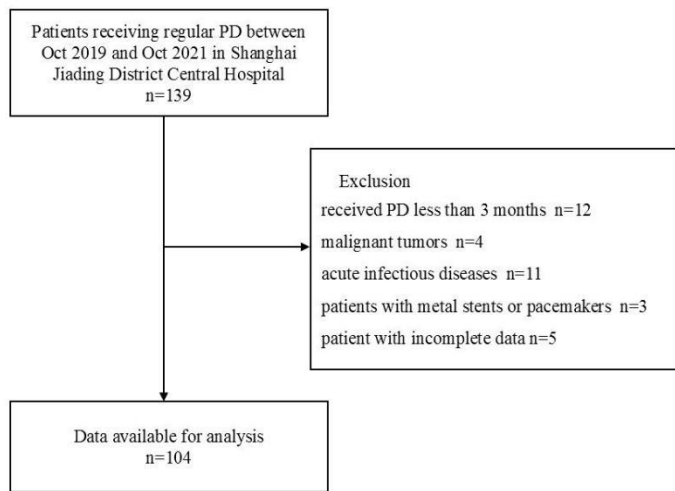

Supplement: Supplemental Material [file IRNF_A_2113794_SM4614.pdf]
